# 中空纳米材料的制备及其在样品前处理中的应用进展

**DOI:** 10.3724/SP.J.1123.2022.09027

**Published:** 2023-06-08

**Authors:** Xuemei WANG, Lixia HUANG, Na YUAN, Pengfei HUANG, Xinzhen DU, Xiaoquan LU

**Affiliations:** 甘肃省生物电化学与环境分析重点实验室, 生态功能高分子材料教育部重点实验室,西北师范大学化学化工学院, 甘肃 兰州 730070; Key Laboratory of Bioelectrochemistry and Environmental Analysis of Gansu Province, Key Laboratory of Eco-functional Polymer Materials of Ministry of Education, College of Chemistry and Chemical Engineering, Northwest Normal University, Lanzhou 730070, China

**Keywords:** 中空纳米材料, 样品前处理, 应用, 研究进展, hollow nanomaterials, sample pretreatment, application, research progress

## Abstract

样品前处理技术在复杂样品(如生物、食品和环境等样品)的分析过程中起着至关重要的作用,是整个分析过程的关键,其主要目的是使待测物与样品基质或样品中的干扰物质分离,并达到仪器可以分析检测的状态。对于样品前处理技术而言,有着优异吸附性能的吸附剂是核心和关键,因此开发具有高选择性和高富集性的吸附材料是该技术目前面临的最大挑战。近年来,各类性能优异的纳米材料被应用于样品前处理领域,发展了众多具有功能多样化、高选择性、高富集性的纳米萃取材料。中空纳米材料是一类在固体壳内具有很大空隙的纳米粒子,因有较大的有效表面积、丰富的内部空间、短的质量传输路径和较高的承载能力等优点,在样品前处理领域表现出了巨大的应用潜力,其主要是通过*π-π*堆积、静电、氢键以及疏水作用等相互之间的协同作用实现对目标分析物的高效分离和富集。同时由于中空纳米材料优异的物理化学性能,也获得了各个研究领域的广泛关注,成为材料科学的研究前沿。但是,中空纳米材料的合成方法仍存在步骤复杂、成本较高、条件相对严苛、涉及剧毒物质等问题。本文总结了中空纳米材料的主要类型、合成方法以及在样品前处理中的研究进展,探讨了中空纳米材料在合成方法上遇到的挑战。最后,对中空纳米材料在样品前处理中的应用及发展进行了展望。

## Abstract (en)

Sample pretreatment technology plays a vital role in the analysis of complex samples and is key to the entire analytical process. Its main purpose is to separate the substance to be measured from the sample matrix or interfering substances in the sample and to achieve a state in which the instrument can be analyzed and detected. Traditional sample pretreatment techniques include liquid-liquid extraction, liquid-solid extraction, precipitation separation, solvent volatilization-rotary evaporation, filtration, and centrifugation. However, the applications of these methods are limited by their low extraction efficiency, complicated operation, long time consumption, unstable recovery, use of large amounts of organic solvents, and large error rates. Several new sample pretreatment techniques, including solid-phase extraction, magnetic solid-phase extraction, solid-phase microextraction, and dispersive solid-phase extraction, have been developed and rapidly applied to various fields to overcome the shortcomings of traditional sample pretreatment methods. However, the development of adsorbent materials with high selectivity and enrichment capability remains a challenge in sample pretreatment technology, in which adsorbents with excellent adsorption performance are crucial. In recent years, various nanomaterials with remarkable properties have been introduced and applied to sample pretreatment, and numerous nano-extraction materials with diverse functions and high selectivity and enrichment capability have been developed. Hollow nanomaterials are nanoparticles with large voids in their solid shells. Owing to their advantageous properties, which include a large effective surface area, abundant internal space, low density, variety of preparation methods, structural and functional tailorability, short mass transmission path, and high carrying capacity, hollow nanomaterials show great application potential in sample pretreatment. The extraction mechanism of these materials is based on the synergistic effects of *π-π* stacking, electrostatic, hydrogen-bonding, and hydrophobic interactions to achieve the efficient separation and enrichment of the target analytes. Given their noteworthy physicochemical properties, hollow nanomaterials have gained wide attention in various research fields and are considered a research frontier in the field of materials science. Changing the structure or surface properties of the core and shell can lead to various hollow nanomaterials with unique properties. Such changes can create synergy between the physicochemical properties and structural function of the original core-shell material, leading to novel materials with superior performance compared with the starting materials and broad application prospects in sample pretreatment. Nevertheless, only a few hollow nanomaterials with diverse structures and functions are currently used for sample pretreatment, and their adsorption capacity for target analytes is often unsatisfactory. Consequently, enhancing the adsorption selectivity of these materials toward various analytes is the most important step in sample pretreatment. First, hollow nanomaterials with a large specific surface area and suitable pore size can be designed to achieve the specific adsorption of target analytes of varying sizes. The combination of hollow nanomaterials with other materials presenting desirable adsorption properties could also lead to synergistic effects and enhance the performance of composite hollow nanomaterials. In addition, more green methods to prepare hollow nanomaterials with outstanding selectivity can be explored to achieve the superior adsorption of a specific target analyte. Efforts to synthesize hollow nanomaterials have been met with great success, but the available synthesis methods still suffer from complicated steps, high costs, relatively harsh conditions, and the use of highly toxic substances. This paper summarizes the main types of hollow nanomaterials, their synthesis methods, and research progress on sample pretreatment technologies (solid-phase extraction, solid-phase microextraction, magnetic solid-phase extraction, and dispersive solid-phase extraction) and describes the challenges encountered in the synthesis of hollow nanomaterials. The applications and developments of hollow nanomaterials in sample pretreatment are also discussed.

样品前处理技术在分析化学中具有举足轻重的地位,它决定着分析化学发展的快慢。在整个分析检测过程中,样品前处理是最为关键的一步,其在环境、食品、生物、药物分析等领域起着重要的作用^[1]^,其实质是从复杂样品基质中提取、净化、分离、富集目标分析物的过程,目的是将待测组分转化成可测定的形式,从而进行定性、定量的分析检测^[2,3]^。新型的样品前处理技术主要包括固相萃取(solid-phase extraction, SPE)、磁性固相萃取(magnetic solid-phase extraction, MSPE)、固相微萃取(solid-phase microextraction, SPME)、分散固相萃取(dispersive solid-phase extraction, DSPE)等,它们在复杂样品的前处理中发挥了重要作用^[4⇓⇓-7]^。在这些前处理技术中,吸附材料的选择是关键因素,因此开发选择性好、富集能力强的吸附剂是该技术的最大挑战。

中空纳米材料(hollow nanomaterials, HoMs)是指具有内部空洞和明确边界的纳米材料,由于其具有较大的比表面积、较低的密度、短的传输路径、高负载能力等突出特性,因此被广泛应用于吸附、环境修复、催化、传感、药物输送等领域^[8⇓-10]^。与实心纳米材料相比,因其原有材料的理化特性和结构功能之间可以产生协同作用,因此在特定的应用中可以展现出比实心纳米材料更加优越的性能。所以,对于中空纳米材料而言,结构的合理设计和可控合成不仅对实现材料在特定应用中的性能有非常重要的作用,而且对结构-材料-功能的构效关系认知也具有重要意义^[11]^。基于HoMs有更加优异的性能,所以HoMs十分适合用作吸附剂,在样品前处理领域表现出巨大的发展潜力。本文总结了近年来HoMs的主要分类、合成方法以及在样品前处理方面的应用和评述,并对其发展前景进行了展望。

## 1 中空纳米材料的分类

根据HoMs核壳层数的不同,可分为单层中空纳米材料、核壳中空纳米材料和中空多壳纳米材料。

### 1.1 单层中空纳米材料

单层中空纳米材料即内部中空无核,仅由外层的壳层所组成的中空结构。由于这种结构具有表面积较大、密度较低和孔容较大等优点,在储能和催化等领域具有广泛的应用前景^[12,13]^。Wang等^[14]^通过在乙二醇-乙醇混合溶液中对乙酸锌进行溶剂热处理,制备了C掺杂的ZnO中空微球(BHM)(图1)。乙二醇(EG)的存在促使了单层空心球的形成,通过控制反应时间将其转化为具有可调壳厚和空穴空间均匀的BHM,并将其用作有机污染物降解的光催化剂和光电化学(PEC)水分解的光阳极材料。结果表明,具有空心结构和C掺杂的ZEG-12(经过12 h溶剂热处理的ZnO)具有最佳的光吸收能力,ZEG-12表现出约5%的电催化活性。当用作降解有机污染物的光催化剂和PEC水分解的光阳极材料时,活性分别比原始ZnO纳米颗粒高8.9倍和10.5倍。此外,许多不同性质的单层中空纳米材料也相继被报道,如C、Fe掺杂的BiOBr、NiCo_2_O_4_、BiOCl、SnO_2_、CuO、ZrO_2_、C/Fe_3_O_4_、V_2_O_5_、ZnFe_2_O_4_、TiO_2_等。虽然这种单层中空纳米材料现被广泛应用在各个领域,但目前还存在结构和组成较为简单、调控方式比较单一等缺点。

**图1:**
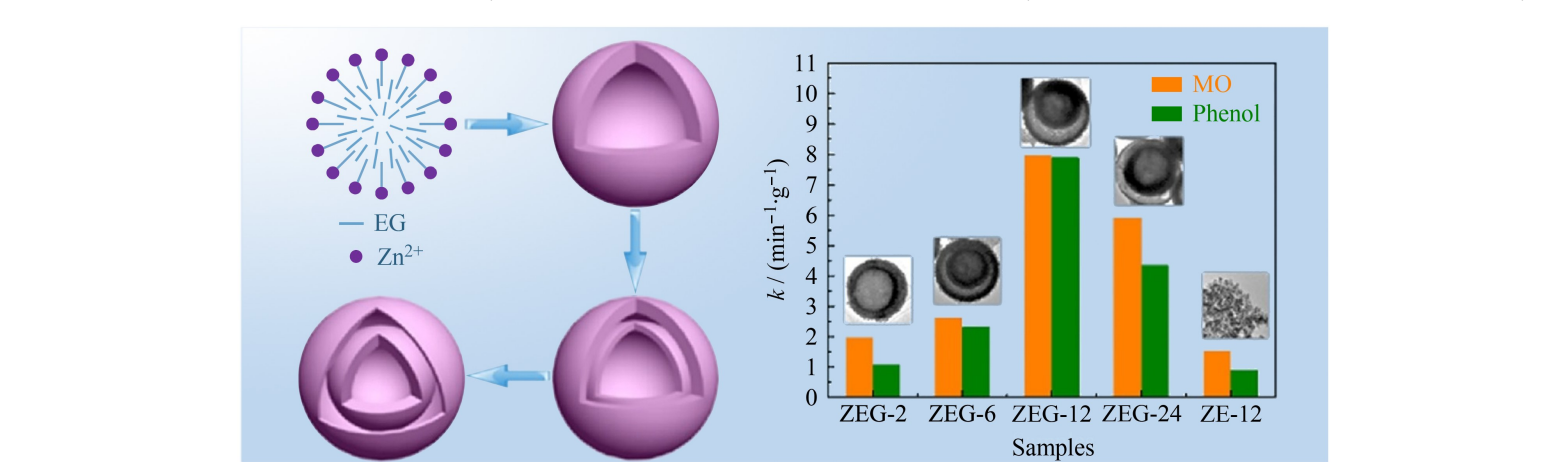
采用溶剂热法合成壳层厚度和空穴空间可控的C掺杂ZnO微球^[14]^

### 1.2 核壳中空纳米材料

核壳中空纳米材料是由外面的壳和里面的核所组成的纳米材料,与单层中空纳米材料相比,其内部有核,可以同时结合核和壳的功能,使其性能更加优异。在合成过程中,核和壳都具有可调节性,通过控制实验条件就可得到不同性能的核壳中空纳米材料^[15⇓-17]^。核壳中空纳米材料可分为两种,一种为核壳紧贴的结构,这种结构的核和壳是紧密贴合的,中间没有空隙,内部存在较大的空腔,会赋予材料更大的比表面积;另一种为核壳分离结构,核、壳之间存在空隙,其结构类似于蛋黄-蛋壳结构,具有该结构的中空纳米材料由于其独特的空腔和内核,可以赋予材料更多的功能,而不损失材料自身的性能,结合了核壳结构和中空结构两类的优点^[18]^。Xiong等^[19]^通过层层自组装合成了具有特定核壳结构的新型吸附剂Fe_3_O_4_@Carbon@ZIF-8(磁性碳@沸石咪唑酯骨架-8)(图2), 其中亲水性碳质壳在Fe_3_O_4_和ZIF-8之间起着连接剂和稳定剂的作用,该材料不仅具有高的比表面积,还具有丰富的官能团可以与金属离子和染料分子相互作用。将该材料用于选择性吸附复杂废水中的刚果红(CR)和Cu(Ⅱ),对CR和Cu(Ⅱ)的吸附容量分别为806.45 mg/g和234.74 mg/g。该复合材料对CR的吸附机理主要为静电作用、氢键和*π-π*键,对Cu(Ⅱ)的吸附机理主要为静电作用、离子交换和微化学沉淀等。因此核壳纳米材料Fe_3_O_4_@Carbon@ZIF-8是一种高效磁性吸附剂,具有良好的稳定性和可重复性,适用于复杂废水中CR和Cu(Ⅱ)的选择性吸附。除此之外,许多核壳结构的中空纳米材料被开发出来,核壳型的中空材料如Fe_3_O_4_@SnO_2_、Fe_3_O_4_@MIL-100、Fe_3_O_4_@COF、*α*-Fe_2_O_3_@CeO_2_、Fe_2_O_3_CeO_2_@MnO_2_中空微球等,蛋黄壳的中空材料如ZIF-67@Co-LDH SiO_2_、Fe(OH)_3_@C中空微球、ZnCo_2_O_4_中空微球、LaFeO_3_中空微球、MoS_2_/C纳米球、立方形态的GNA@AuAg核壳纳米颗粒等。

**图2:**
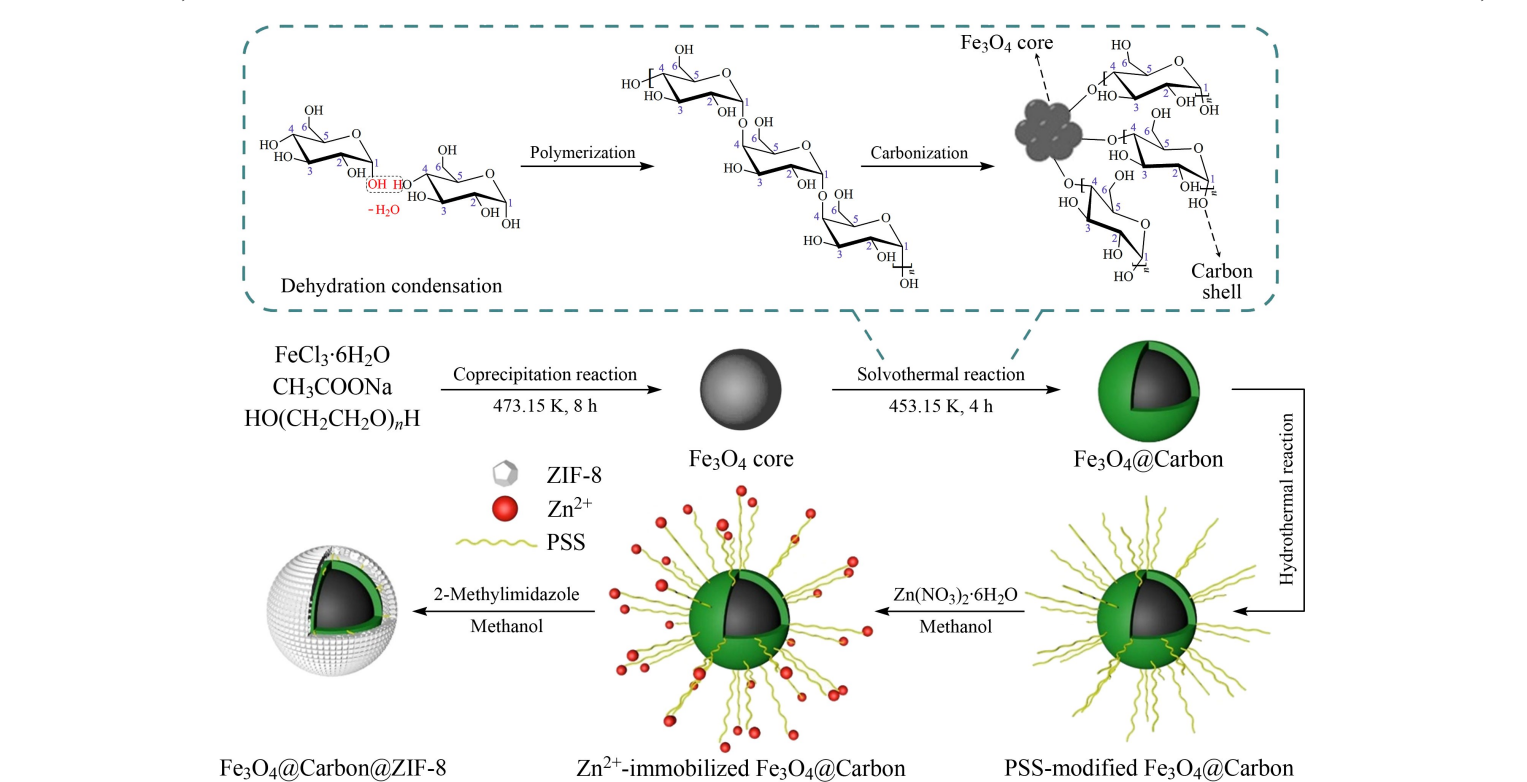
核壳纳米颗粒Fe_3_O_4_@Carbon@ZIF-8的合成路线^[19]^

### 1.3 中空多壳纳米材料

与单壳层的纳米材料相比,多壳层纳米材料壳间距的存在使其具有更强的优势,因此多壳层中空纳米材料的合成越来越受到重视,通过对单壳层中空纳米材料的表面进行改性处理,可得到具有多壳层结构的中空纳米材料。多壳层中空纳米材料即为内部中空无核,外层是由多个壳层相互独立、层层嵌套而组成的中空多壳层结构。多壳层结构的纳米材料具有多个界面,通过控制其组成成分,每个界面甚至可以产生不同的功能,从而精确控制化学反应环境^[20⇓-22]^。与单层和核壳中空纳米材料相比,多壳层中空纳米材料具有更大的比表面积、低密度、短的传输路径等优点,因而应用更加广泛。Dong等^[23]^用硬模板法制备了五壳层的SnO_2_空心微球(图3), 将其用作光电阳极构建染料敏化太阳能电池(DSSC),由于增强了光散射而显示出非常高的光转换效率。理论模拟和P25(锐钛矿晶和金红石晶混合相的二氧化钛)薄膜上多壳层SnO_2_-CDS散射层组成的复合光电阳极的实验结果表明,该复合光电阳极的总转换效率为9.53%,与P25相比,增加了30%以上。由于中空多壳纳米材料的快速发展,众多该结构的材料已被成功制备。如多壳层TiO_2_中空微球、Co_3_O_4_中空微球、C中空微球、CuO@NiO中空微球、TiO_2_/Fe_2_TiO_5_异质中空微球、NiCo_2_S_4_@NiFe中空微球、LiMn_2_O_4_中空微球、Au/CeO_2_中空微球、NiCo_2_O_4_中空微球以及多壳层的空心立方体ZnO等。

**图3:**
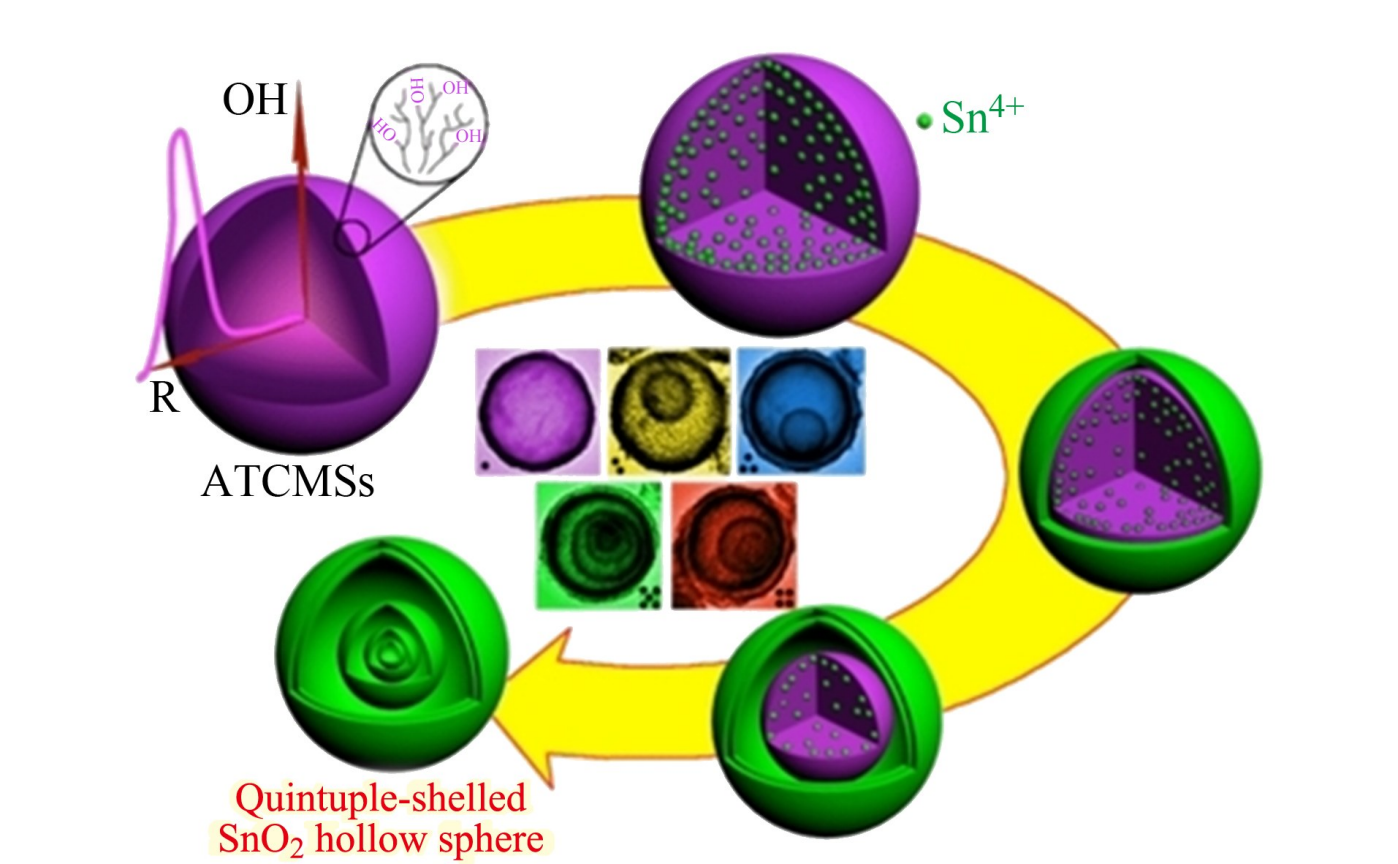
五壳层SnO_2_空心微球形成过程示意图^[23]^

## 2 中空纳米材料的制备方法

在过去的几十年里,中空纳米材料的合成和应用都取得了巨大的进展,且在样品前处理以及其他领域都表现出良好的性能。随着对这种具有高性能材料的需求增多,所需中空纳米材料的合成方法就处于一个相当重要的地位。因此有必要对合成方法进行系统总结,为未来的发展提供一些参考。中空纳米材料的合成方法主要有无模板法、软模板法、硬模板法、自模板法等。

### 2.1 无模板法

无模板法即不使用任何模板剂合成中空纳米材料,在整个过程中不涉及模板的制备和去除,因此使用该方法制备中空纳米材料时有时间短、制备成本低、步骤简便等优点。目前,无模板法主要有以下3种:定向附着、奥斯特瓦尔德(Ostwald)熟化和柯肯达尔效应。

定向附着的本质是晶体沿着相同的晶体学方向生长发展,附着形貌主要由晶体的原始形貌以及晶体的稳定性所决定^[24]^。如Xu等^[25]^在研究单晶Cu_2_O多壳空心球形成过程的基础上,提出了单晶空心球的生长机理主要是定向附着。经过表征发现,粒径为2~5 nm的Cu_2_O空心纳米粒子,在十六烷基三甲基溴化铵(CTAB)多层囊泡的方向下首先通过定向附着形成结晶良好的多孔壳空心,然后通过Ostwald熟化使多孔壳进一步结晶并致密,形成结晶良好的空心球。Sun等^[26]^通过水热合成法制备了中空异质结构的纳米材料钒酸铋(m-BiVO_4_)(图4),该材料的形成过程主要是依赖定向附着和Ostwald熟化。通过光催化降解罗丹明B(RhB)方法分析其性能,该材料由于具有中空结构的优点而表现出较高的催化活性、稳定性以及耐久性,经过四轮循环光降解RhB后,所制备的中空纳米材料的光催化能力没有出现任何下降。

**图4:**
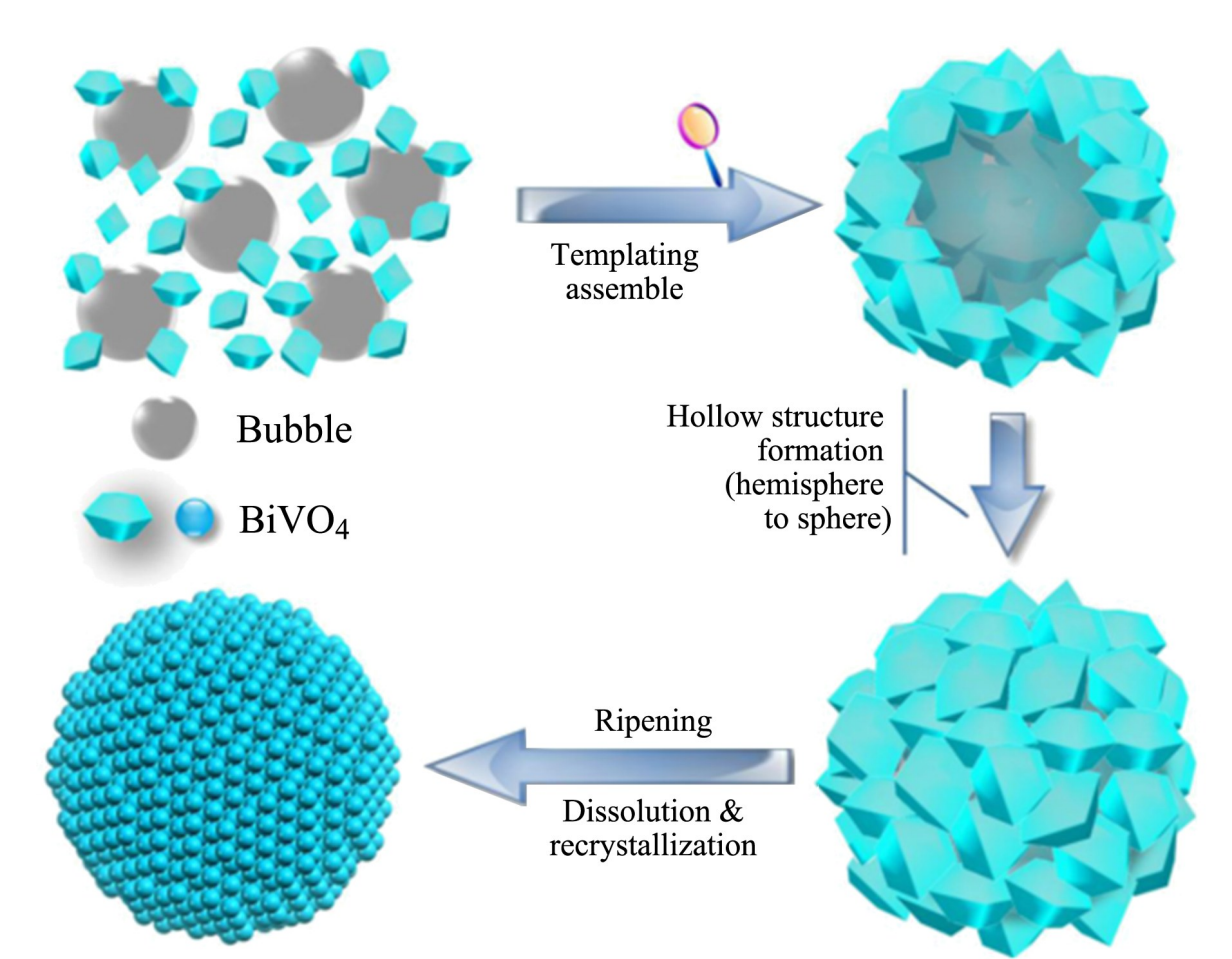
m-BiVO_4_的形态演变示意图^[26]^

Ostwald熟化是一种在固溶体或液溶胶中观察到的现象,描述了一种非均匀结构随时间流逝所发生的变化:溶质中较小型的结晶或溶胶颗粒溶解,并再次沉积到较大型的结晶或溶胶颗粒上^[27]^。使用该方法制备中空纳米材料时,中空结构的主要形成机理是在最开始的固体聚集物内部存在着固有密度变化,早期形成的纳米晶体的聚集/组装过程基本上是动态且缓慢的,从而形成了一个由密集填充的核和松散填充的壳组成的中间结构。核壳结构保证了两种Ostwald熟化的发生,即向内和向外成熟,最终形成多层空心结构。该过程通常使用有机溶剂或混合溶剂,以降低反应初期形成的纳米晶的组装速率。如Nikhila等^[28]^通过无模板溶剂热法在常温条件下制备了中空多壳TiO_2_纳米球,其形成机理主要是Ostwald熟化和重结晶;使用透射电子显微镜(TEM)表征不同反应时间的产物,分析各个阶段的形貌转变。结果表明空心球体的形成虽然经过了各种复杂的内部变化,但仍然保留了单晶性质。Guo等^[29]^基于Ostwald熟化的现象过程,用无模板溶剂热法合成了中空TiO_2_微球,因有机溶剂的性质对材料的微观结构和性质有着显著的影响,所以该工作探究了溶剂对该材料形成的影响(图5)。以甲醇、异丙醇、乙二醇和丙酮等4种有机溶剂与乙醇混合作为混合溶剂合成中空TiO_2_微球,由于丙酮具有中等极性、低沸点和黏度小等特性,对中空结构的构建帮助最大。为了评价不同溶剂体系制备的中空TiO_2_纳米微球的光催化活性,测试了样品的紫外/可见吸收。实验结果表明,具有中空纳米结构的TiO_2_微球具有更大的吸收面积,这意味着其具有更强的捕光能力和光催化性能。因此,以RhB作为有机染料,进一步评价了中空TiO_2_纳米微球的光催化活性,当用紫外线照射120 min时,RhB溶液的残留率几乎为零,说明具有中空结构的TiO_2_纳米微球对RhB的吸附能力更强。

**图5:**
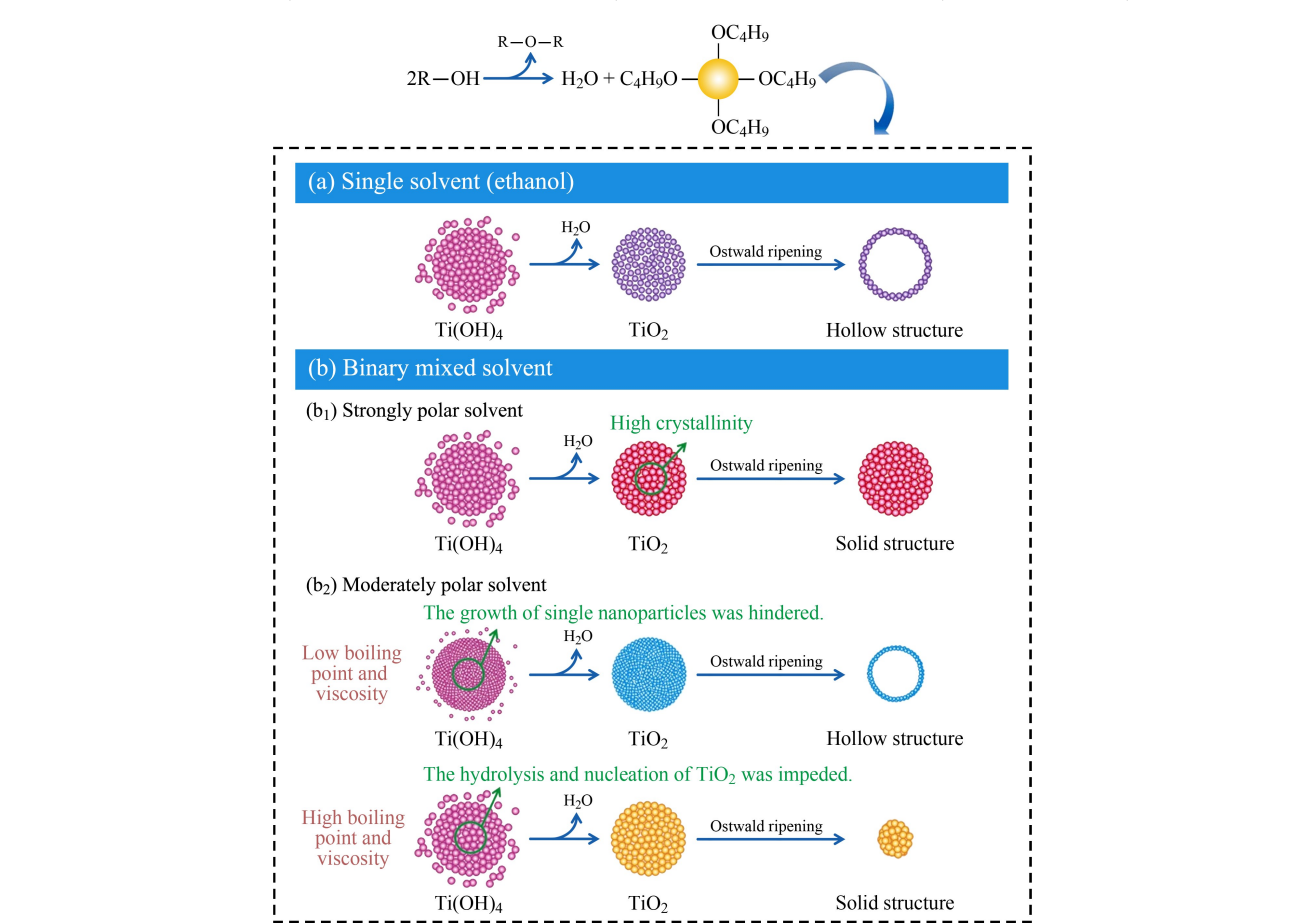
所制备的TiO_2_纳米结构的溶剂特性和形态行为之间的相关性示意图^[29]^

柯肯达尔效应是指两种扩散速率不同的金属在扩散过程中会形成缺陷,从而导致空心结构的产生,形成空心结构材料。在利用柯肯达尔效应制备中空结构的纳米材料时,通常采用核壳结构的材料作为前驱体,选择堆芯和壳层的组成,利用两者扩散速度的不同产生空穴,从而合成中空的内部^[30]^。Park等^[31]^首次通过柯肯达尔效应成功制备了中空纳米材料NiO(图6)。将NiSe_2_纳米八面体作为前体,在500 ℃氧化的过程中,NiSe_2_中镍离子和硒组分向外扩散比氧气的向内扩散更快,这导致硒化物通过柯肯达尔效应完全转化为中空纳米材料NiO。将该材料作为锂离子电池(LIB)的阳极来探究其性能,结果表明其因具有大量的内部空隙而有优异的电化学性能。

**图6:**
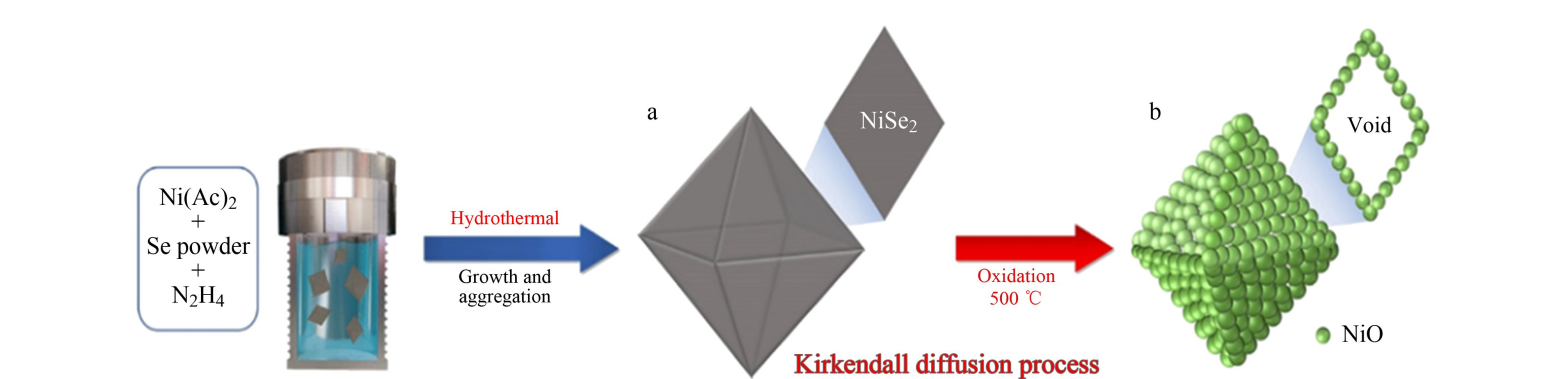
独特中空纳米材料八面体NiO的形成机理示意图^[31]^

### 2.2 软模板法

用软模板法制备中空纳米材料时,主要以气泡、液滴、大分子胶束或者聚合物囊泡等比较软的物质作为模板,之后软模板、溶剂以及前驱体之间相互作用,产生目标材料的外壳,然后晶体在这些模板和溶液的界面处聚集生长,最后去除模板,即可得到所需要的目标产物中空纳米材料^[32]^。值得注意的是,用软模板法制备中空纳米材料时,会受到如温度、压力、溶液pH值和浓度、无机添加剂种类等诸多因素的影响,因此需要在不同的实验环境条件下进行大量的实验探究,以寻求最佳的反应条件。如Li等^[33]^通过简便的软模板溶剂热法,成功合成了一种独特的具有多孔表面的双壳ZnO中空微球(图7)。

**图7:**
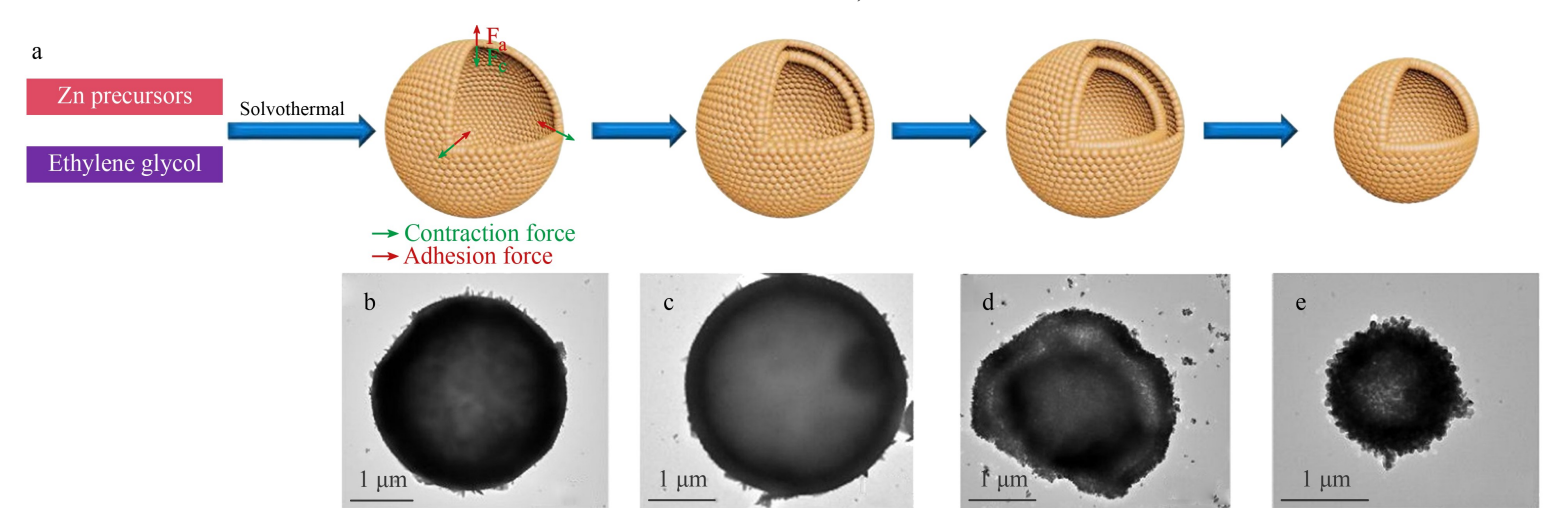
(a)ZnO空心微球的形成示意图与(b-e)ZnO空心微球在溶热时间分别为2、6、12、24 h的演变过程^[33]^

具体做法为将乙二醇作为软模板,促使初始单层空心微球的形成,接着控制反应时间,获得具有可调壳数和空隙空间的双壳ZnO中空微球,将其用作检测丙酮的传感材料来评价其性能。实验结果表明,双壳ZnO中空微球作为传感材料时,传感器对100 mg/L丙酮表现出高响应,且其性能优于ZnO微粒和单壳ZnO中空微球。此外,该传感器具有检出限低、工作温度低、对丙酮有高选择性和长期稳定性等优点。Shao等^[34]^基于软模板法,采用一锅法合成了一种新型金属氢氧化物/氧化物中空纳米材料。该材料的合成策略主要在于控制壳的沉积、生长和结晶之间的反应动力学与水热系统中过热水对SiO_2_核的同时蚀刻之间的平衡来得到目标产物。采用该方法合成了具有可调结构、形状和壳厚度的羟基氧化铁中空纳米材料((FeOOH)HNs),同时也通过简单地改变金属前驱体和反应温度,成功合成了几种重要的金属氧化物中空纳米材料,如*α*-Fe_2_O_3_、CeO_2_、SnO_2_和TiO_2_。选择不同厚度和壳结构的(FeOOH)HNs作为光催化剂来降解RhB,以便分析该材料的性能,结果表明,所合成的具有分层和较厚壳的(FeOOH)HNs表现出优异的光催化活性和出色的稳定性,从实际应用来看,该材料是光催化降解水中有机污染物较好的候选材料。

### 2.3 硬模板法

硬模板法是合成中空纳米材料最常用的一种方法,它主要分为次序模板法(顺序模板法,STA)和非次序模板法(层层自组装法)两大类。用硬模板法制备中空纳米材料主要有以下步骤:(1)合成模板;(2)修饰模板以获得良好的表面性能;(3)将所制备的材料或前驱体涂层沉积在模板上;(4)通过蚀刻或热解等方法选择性地去除模板。在上述步骤中,步骤(3)最为关键。如果目标材料与模板相容性好,表面修饰步骤也可以省略^[35]^。硬模板法具有操作简单、可以精确调控中空纳米材料的形貌结构等优点,因此可以制备不同结构、性能、形貌的中空纳米材料。用该方法合成中空纳米材料时,对于模板的选择也至关重要,它决定着合成材料的性能优异性,可以作为硬模板法材料的有金属纳米颗粒、金属氧化物、金属氢氧化物、金属板聚合物、SiO_2_、聚苯乙烯、碳球、苯二酚/甲醛树脂(RF)微球等。

#### 2.3.1 顺序模板法

顺序模板法是王丹课题组在2009年提出的制备中空多壳结构材料的一种方法^[36]^。其本质是在模板去除的过程中,模板上富集的前驱体聚集成壳与模板的分解收缩同时进行,模板因体积的逐渐收缩存在一个动态的变化过程,因而能够依序多次发挥模板作用逐步形成中空多壳结构。Yu等^[37]^以蔗糖为碳质球源,合成了氧化铜(CuO)和氧化亚铜(Cu_2_O)的复合空心球,而非纯CuO空心球。通过精确地控制前体煅烧速率,可以从单壳、双壳到三壳空心球调整产品的结构(图8)。

**图8:**
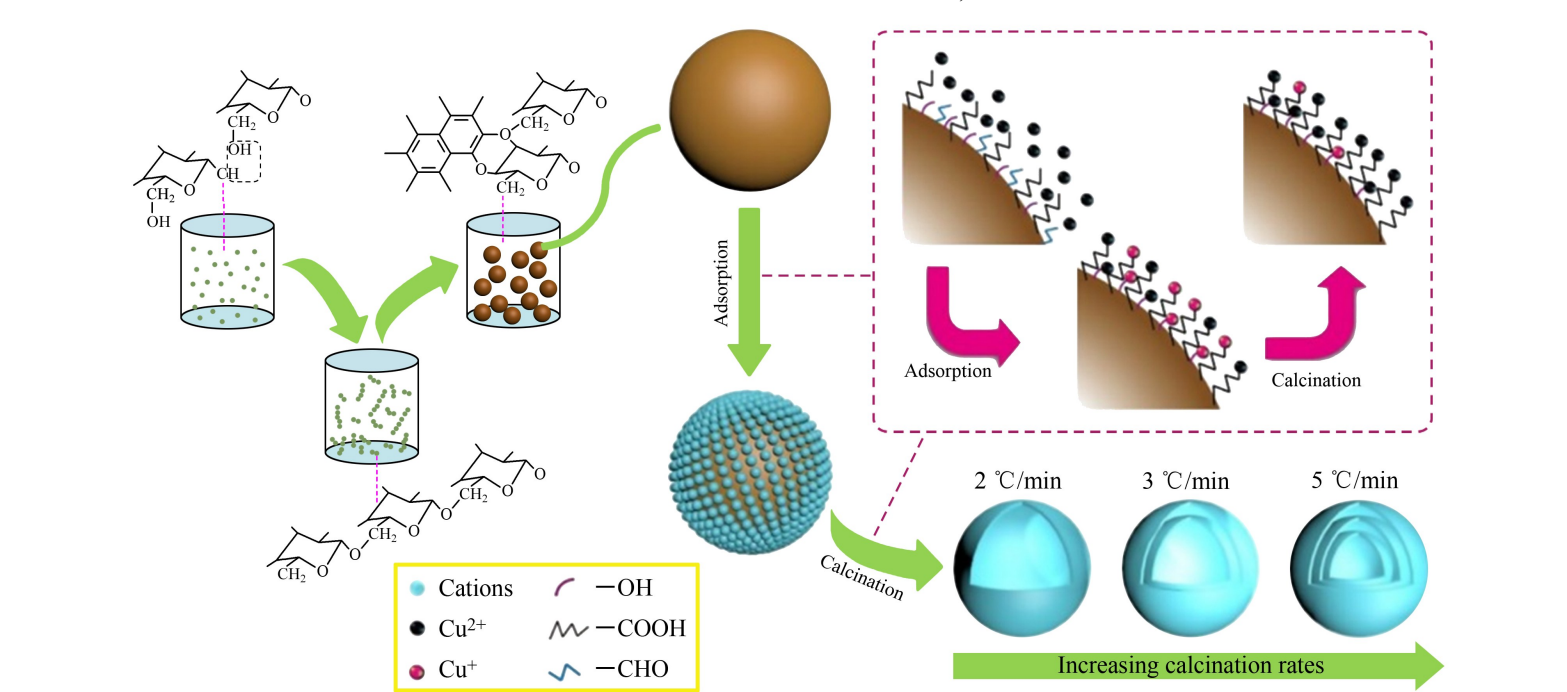
多壳氧化铜空心球的形成示意图^[37]^

纯CuO空心壳球具有约900 nm的均匀直径和45 nm的平均壳厚度,而Cu_2_O/CuO复合空壳球的直径和厚度分别为90~500 nm和15~35 nm。这两种空心球作为气体传感器对乙醇气体的响应都有很大的提高,三壳CuO/Cu_2_O空心球在140 ℃对1000 mg/L乙醇气体表现出最高的响应。Wang等^[38]^采用顺序模板法合成了中空多壳结构TiO_2-_*_x_*作为锂硫电池的硫载体,实现了多硫化物的双重物理化学吸附,从而显著提高了锂硫电池的库仑效率和循环稳定性。特别是基于中空多壳结构TiO_2-_*_x_*,不仅可以通过物理方式捕获多硫化物,还可以通过氧空位和Ti^3+^在壳层表面的强结合作用以化学方式锚定多硫化物。结果表明,该材料有着优异的电化学性能,比容量较大,库仑效率较高,在1000次循环中仍保持79%的容量。作为锂硫电池的硫载体,为长循环锂硫电池的发展注入了活力。

#### 2.3.2 层层自组装法

层层自组装法主要是以聚苯乙烯球或二氧化硅球等为模板进行层层包覆,之后通过刻蚀过程去除模板获得中空多壳纳米材料,此方法步骤较多,但是在合成过程中比较容易控制材料的形状和层数。Chen等^[39]^首次通过溶胶-凝胶工艺的逐层组装成功制备了双壳SiO_2_/TiO_2_中空纳米管,该材料具有大的表面积和高的孔体积,中空纳米管的内壳厚度和外壳厚度分别约为80 nm和120 nm。在制备过程中,SiO_2_和TiO_2_的壳厚度是可控的,主要取决于制备它们的模板正硅酸四乙酯(TEOS)和钛酸四丁酯(TEOT)的浓度,该制备方法可以扩展到制备其他介孔双壳结构和其他尺寸的空心结构。Chen等^[40]^对具有特殊性能的蒙脱石材料进行了新的研究,主要通过基于单模板的层层自组装法制备了蒙脱土空心和分级介孔微球(MMTNS@CS-HMPHS)(图9)。

**图9:**
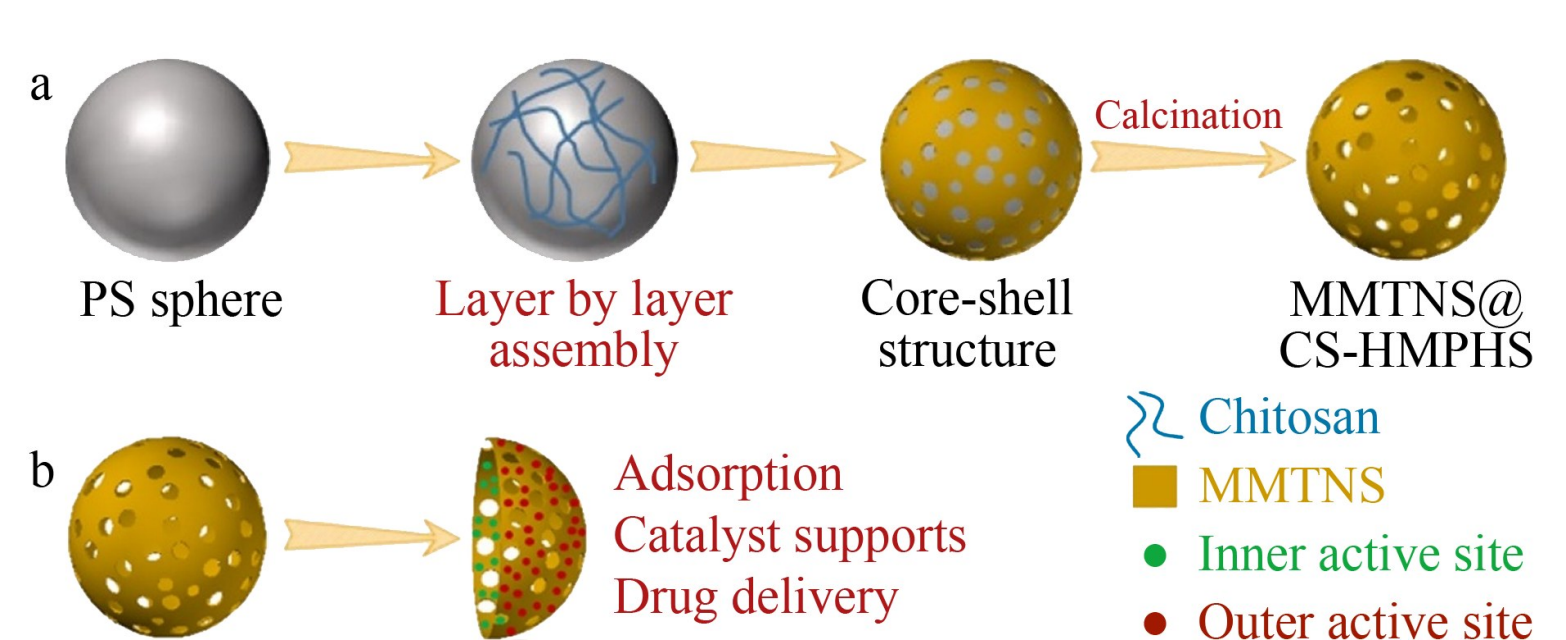
(a)MMTNS@CS-HMPHS的合成过程示意图及其(b)独特微观结构和潜在应用^[40]^

对该复合材料的形貌和表面性质等进行了表征,高的比表面积主要得益于MMTNS独特的层状结构,及在MMTNS@CS的壳体上形成的介孔通道。此外,该复合材料的表面官能化和孔径大小由于层层自组装方法有着特定的属性,易于调整。最后,除了独特的微观结构之外,该复合材料还具有较好的活性中心,大大地提高了其在吸附、药物输送和催化剂载体等领域的性能。

### 2.4 自模板法

用自模板法合成中空纳米材料时,其自身在合成材料中是杂质,为了获得所需的最终产物,还需要利用酸解或者煅烧去除模板。该方法一般需要两步来合成,第一步是制备模板材料,第二步是将模板材料转化为中空结构,使模板材料完全或者部分并入壳中^[41]^。与硬模板和软模板方法相比,该方法中使用的模板不仅提供了创建中空内部的空间,而且也是构建外壳的材料来源。根据化学转化过程,外壳的材料可以与模板材料或模板材料的衍生物相同。自模板法的优点在于具有相对简单的合成步骤、高重复性、低生产成本以及对壳厚度和颗粒均匀性的良好控制。最后,使用自模板法合成中空纳米材料时,该过程通常涉及化学转化反应,这些反应可以更好地帮助我们理解纳米材料的合成机理。Wang等^[42]^通过自模板法以MnO_2_为前驱体合成了具有中空结构和硫化物涂层的富锂层状正极纳米材料Li_1.2_Mn_0.6_Ni_0.2_O_2_(LMNO),并将其作为电池的阴极材料探究其性能(图10)。结果表明,Ni-Mn硫化物均匀分布在阴极表面,硫化过程中扩大的中空结构可以缩短锂离子扩散路径,并适应电池工作期间的体积变化。Ling等^[43]^采用简单溶剂热自模板法合成核壳纳米复合材料SiO_2_@MnSiO_3_,并对其催化降解能力和对重金属离子的去除能力进行了探究。该工作主要通过对SiO_2_纳米球、MnCl_2_·4H_2_O、乙二胺(EDA)和乙二醇进行简单的一步热处理,在EDA存在下,乙二醇蚀刻自模板SiO_2_纳米球,导致硅酸盐阴离子从SiO_2_球体表面缓慢释放,并且在界面区域内发生Mn^2+^和硅酸盐阴离子之间的快速沉淀反应,最终导致SiO_2_纳米球体表面形成MnSiO_3_壳。对其性能进行探究,发现SiO_2_@MnSiO_3_对亚甲基蓝(MB)的氧化降解表现出高催化活性,超过93%的MB可在40 min内分解。此外,它还可作为高效去除水溶液中Pb^2+^的潜在吸附剂,对Pb^2+^的吸附容量高达50.5 mg/g,吸附容量显著高于其他常规吸附剂。用自模板法合成中空纳米材料的方法不仅简便、高效,而且可扩展到制备其他中空纳米复合材料。

**图10:**
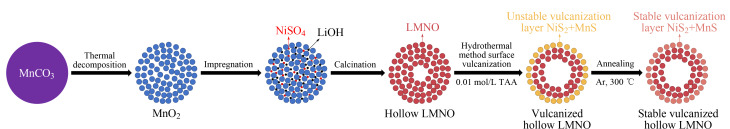
制备稳定的硫化空心LMNO的示意图^[42]^

## 3 中空纳米材料在样品前处理方面的应用

目前,中空纳米材料的合成研究正处于快速发展的阶段,许多性能优异、应用潜力较大的中空纳米材料已被合成,这为中空纳米材料在样品前处理技术中的应用研究提供了丰富的来源和选择。由于中空纳米材料具有独特的结构、超高的比表面积、低密度和可控孔径分布等特点,因此有着更加丰富的活性位点和极高的吸附负载量,其主要通过与目标分析物之间的*π-π*作用、疏水相互作用、氢键作用等使目标分析物被高效分离和富集。因此,中空纳米材料作为新型吸附剂在样品前处理技术中受到了广泛的关注。

### 3.1 固相萃取

SPE是20世纪80年代中期开始发展起来的一种样品前处理技术,它主要用于样品的分离、纯化和浓缩,与传统的液液萃取法相比有许多的不同,它可以更加有效地将分析物与干扰组分分离,减少样品预处理过程,操作简单,所需要的时间少,可以提高分析物的回收率,被广泛地应用在医学、制药、食品、环境、化工等领域^[44,45]^。SPE的主要原理是利用固体吸附剂将液体样品中的目标化合物进行吸附,有效地将样品的基体和干扰组分分离,接着用洗脱液进行洗脱,从而达到分离和富集目标化合物的目的。这种萃取方法在很大程度上增强了对分析物的检出能力,提高了分析物的回收率,因此受到了人们广泛的关注而迅速发展起来^[46]^。如Qin等^[47]^以聚(苯乙烯-衣康酸酐)颗粒为核心模板,衣康酸酐和反式茴香醚为壳成功制备了一种羧基功能化的中空聚合物微球纳米材料(CHPMs),将该材料作为吸附剂装入固相萃取柱中,并结合电感耦合等离子体质谱联用仪(ICP-MS)来检测食品中的V(Ⅴ)、Cr(Ⅲ)、Cu(Ⅱ)、Cd(Ⅱ)和Pb(Ⅱ)等离子。

该方法具有较宽的线性范围(0.05~15 μg/L)和较低的检出限(0.8~3.2 ng/L),相对标准偏差(RSD)为1.2%~3.5%,回收率在88%以上,有着良好的重复性,因此以羧基功能化的中空聚合物微球作为SPE辅助ICP-MS分析的吸附剂,在复杂样品痕量元素的分析中具有较大的潜力。Yavuz等^[48]^通过溶剂热法合成了海胆状NiCo_2_O_4_空心微球,将其用作SPE的吸附剂,并结合原子吸收法(FAAS)来测定水、食品和街道灰尘样品中的Pb(Ⅱ)。所建立的方法具有较低的检出限,RSD大于2.4%,同时也显示出良好的准确度、精密度和选择性,该吸附剂可重复使用32次。该方法的主要优点是萃取过程可以快速达到吸附平衡,无需涡旋或摇动,省时、环保、简便且成本较低;良好的回收率证明,本研究所建立的方法可以成功地应用于所测样品中铅的分离和预浓缩。Tong等^[49]^通过以聚苯乙烯(PS)为牺牲模板、多巴胺自聚合包覆PS、L-赖氨酸(L-lys)为吸附配体,制备了中空纳米材料L-赖氨酸改性聚多巴胺涂层壳(h-PDA@L-lys)(图11);在制备过程中,PS被蚀刻形成中空结构,可以最大限度地利用位于内外表面的更多结合位点,将该材料作为SPE吸附剂用于从动物肝脏中富集特定胆红素(BR)。在最优条件下,h-PDA@L-lys表现出良好的选择性吸附能力和较高的吸附量;吸附实验表明,该吸附过程为准二级动力学吸附,饱和吸附容量为12.0 mg/g,其优异的吸附容量不仅高于非中空纳米材料的吸附剂,而且还优于许多报道的其他吸附剂^[50]^,该方法已成功应用于动物肝脏中BR的选择性识别。

**图11:**
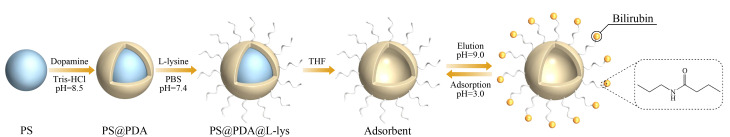
h-PDA@L-lys的合成过程^[49]^

### 3.2 固相微萃取

SPME是1989年由加拿大Waterloo大学Pawlinszyn及其合作者Arthur等提出的一种样品前处理技术^[51]^。传统的样品前处理技术在使用过程中有机溶剂消耗量大,易对环境造成污染,不易操作,且分析速度比较慢。SPME的出现解决了这一系列的缺点。SPME集采样、萃取、浓缩、进样分析于一体,操作方便,耗时短,所用仪器简单,无需其他设备,在操作过程中不仅有机溶剂消耗少、富集能力高,而且灵敏度高,可以实现超痕量分析,达到ng/g级别的检测,适于现场分析,且易于与气相色谱(GC)或高效液相色谱(HPLC)联用^[52,53]^,极大地简化了样品制备步骤,缩短了萃取时间,使得分析结果准确,提高了检测效率。因此,SPME已在环境、食品分析、农业、临床医学等领域被大力推广应用^[54,55]^。

SPME方法主要分为萃取和解吸两个步骤,第一步萃取过程是将具有吸附涂层的纤维放置在样品中进行萃取,接着进行第二步解吸过程,将萃取完的纤维丝插入解吸液里进行解吸,最后进入液相色谱或者气相色谱进行分析。对SPME而言,涂层材料的结构和性质对其萃取性能起主导作用,因此新型SPME涂层材料的开发对实际样品的高效检测起着关键作用^[56,57]^。Hu等^[57]^合成了一种由金属有机骨架(MOFs)衍生的中空碳纳米立方体(HCNCs)新材料作为SPME的纤维涂层材料,制备了一种中空碳纳米立方纤维(HCNCs-F)。通过与气相色谱-质谱联用仪(GC-MS)结合,建立了水样中6种多环芳烃(PAHs)的分析方法,该方法具有线性范围宽(萘为10~2000 ng/L,其他5种分析物为5~2000 ng/L)、重复性好(RSD<8.8%)、检出限低(0.03~0.70 ng/L)和回收率良好等优点;与商业纤维相比,固体碳纳米立方体(SCNC)涂层纤维(SCNCs-F)有着丰富的活性位点、传质短、与目标分析物之间有较强的疏水相互作用等优点,因此对PAHs表现出更优异的富集能力和萃取效率,所建立的方法已成功应用于实际水样中PAHs的测定。Hu等^[58]^还通过用单宁酸(TA)蚀刻沸石咪唑骨架-8(ZIF-8),然后再进行碳化,得到了一种由ZIF-8衍生的双壳中空氧化锌/碳(ZnO/C)纳米材料,将其作为SPME的新型涂层,结合GC-MS建立了复杂环境水样中非极性(苯化合物(BTEX))和极性(氯酚(CPs))污染物的分析方法。该方法具有较低的检出限(BTEX为0.14~0.56 ng/L, CPs为1.10~2.84 ng/L)以及良好的可重复性(0.61%~7.8%),显示出了优异的萃取性能;优异的萃取性能主要是由于中空ZnO/C纳米材料的组成和结构之间的协同作用。在组成上,Zn-OH通过氢键结合PAHs,碳通过*π-π*堆积以及相互疏水作用吸附PAHs。在结构上,基于ZnO/C具有独特的中空双壳结构、丰富的活性位点、壳之间具有空隙空间等,也有利于对PAHs的萃取。

本课题组^[59]^采用配体转化的方法,将ZIF-67的配体2-甲基咪唑用2,5-二羟基对苯二甲酸取代两次后,再进行碳化得到了中空双壳结构的Co_3_O_4_/C纳米材料,将该材料涂覆在螺旋的不锈钢丝表面,作为螺旋固相微萃取(S-SPME)的涂层,制备了一种独特的S-SPME纤维,并结合HPLC对环境水样中的15种PAHs进行分析(图12);所建立的方法具有低检出限(0.002~2.680 μg/L)、宽线性范围(0.005~1000 μg/L)和良好的重复性(RSD为0.7%~10.7%);与商业纤维相比,S-SPME纤维对PAHs表现出优异的富集能力和萃取效率,这可归因于目标分析物与涂层之间存在着强*π-π*堆积作用力、疏水相互作用力。除此之外,涂层材料的中空结构和螺旋纤维等也都可以增加吸附剂与目标分析物的接触面积,从而提高涂层纤维的萃取效率。此方法已成功地用于环境水样中15种PAHs的检测。Xu等^[60]^通过化学沉淀法和碳化法成功制备了核壳结构的Fe_2_O_3_/CeO_2_@MnO_2_微球,并将其用作SPME涂层,用于测定水样中的PAHs。在最优条件下,所建立的方法有着高灵敏度、良好的线性范围、低检出限和优异的重复性,并且单根纤维和5批纤维的RSD分别为4.1%~8.2%和7.1%~11.4%;与商用SPME涂层和其他纳米金属氧化物涂层相比,本研究所制备的SPME涂层对PAHs具有优异的萃取能力,同时核壳微球上产生的丰富表面氧为增强分析物萃取提供了结合位点;该检测方法已被成功地用于测定实际河流水样中的PAHs。

**图12:**
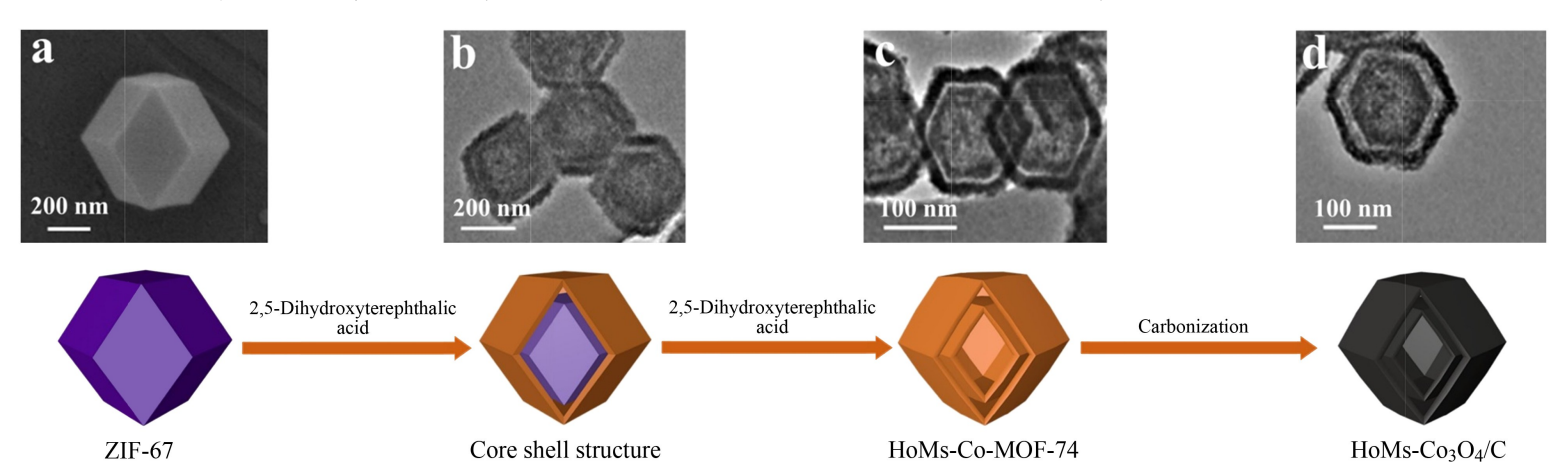
Co_3_O_4_/C中空纳米材料的合成机理^[59]^

### 3.3 磁性固相萃取

MSPE是用磁性纳米粒子或磁性复合材料作为吸附剂的一种样品前处理技术。因传统的SPE有着萃取时间长、萃取一般采用填柱式萃取、洗脱溶剂的体积不可控,还需浓缩和定容、萃取低浓度目标物能力比较弱、抗杂质干扰能力较弱等缺点,很大程度上限制了它的应用^[61,62]^。但是MSPE的产生和发展,从本质上解决了传统SPE的这些缺点,因在使用MSPE的过程中,磁性吸附剂不是填充到吸附柱中,而是直接被加入到样品溶液中,因此它能够在很短的时间内从待测体系中萃取待测物,再通过合适的溶剂将被测物质洗脱下来进行测定^[63⇓-65]^。该样品前处理技术有着所需萃取时间短、有机溶剂用量少、萃取采用的是固相分散萃取、洗脱溶剂的体积可控、只需使用少量的吸附剂就能实现低浓度目标物的萃取、抗杂质干扰能力强、具有较高的萃取效率等优点,这使得它在细胞分离、药物转运、环境科学、食品科学、基因组学、蛋白组学等诸多领域中展现出了广阔的应用前景^[66⇓⇓-69]^。

磁性固相萃取的主要过程分为三部分,首先将所制备的磁性纳米材料加入到样品溶液或悬浮液中,使目标分析物吸附到纳米材料表面;接着将吸附了目标分析物的纳米材料通过外部磁场转移到溶剂中去除杂质;最后将去除完杂质的纳米材料再通过外部磁场转移到洗脱溶剂中洗脱,从而达到分离浓缩目标分析物的作用。本课题组^[70,71]^以金属有机骨架为前驱体,通过一步煅烧法成功合成了多孔笼状中空磁性碳掺杂氧化钴纳米复合材料(CoO@C),将其用作MSPE的吸附剂,并结合HPLC来测定环境水样中的9种PAHs (图13)。该方法的线性范围良好(0.5~1000 μg/L),LOD(0.06~1.30 μg/L)和LOQ(0.19~4.30 μg/L)低,重复性好(RSD为1.1%~6.5%)。与其他商业吸附剂相比,所制备中空磁性纳米复合材料具有优异的吸附能力。Zhou等^[72]^采用简单的水热法合成了核壳型磁性碳微球(Fe_3_O_4_@C),将其作为MSPE的吸附剂,并结合气相色谱-负化学电离质谱法(GC-NCI-MS)来测定环境水样中的多溴联苯醚(PBDEs);在最佳条件下,该方法检出限较低(0.07~0.17 ng/L),线性范围宽(1~1000 ng/L),重复性好(RSD为0.80%~4.58%),用所建立的方法对真实水样中的PBDEs进行了分析,回收率为72.8~97.9%,结果令人满意。Gao等^[73]^以1,3,5-三(4-氨基苯基)苯(TAPB)和2,5-二溴-1,4-苯二甲醛(DBDA)为两个构建单元,在室温下合成了核壳结构的磁性共价有机框架(Fe_3_O_4_@COFs),将其作为二苯胺(DPA)及其类似物的MSPE吸附剂,并结合HPLC测定苹果皮、火柴和湖水中的DPA及其类似物;所建立的方法在0.1~100 μg/mL范围内具有良好的线性关系(相关系数(*R*)>0.9946)、低检出限(0.02~0.08 μg/mL)以及良好的回收率(79.97%~122.52%)。此方法已成功地用于富集和测定苹果皮、火柴和湖水中的DPA及其类似物,表明Fe_3_O_4_@COFs作为新型吸附剂在样品前处理中具有很大的应用潜力。Yang等^[74]^通过自组装氯化磷酸和苯三甲酸制备了一种磁性核壳纳米材料Fe_3_O_4_@MIL-100(MNPs),将其作为MSPE吸附剂,并结合HPLC来测定儿童牙膏中的三氯生。该方法在0.1~50 mg/kg范围内线性关系良好,检出限低(0.03 mg/kg),重复性较好(RSD<5.5%, *n*=4),回收率为90.86%~101.1%,这可归因于MOFs与Fe_3_O_4_的结合增强了MNPs在水溶液中的分散性,在进行MSPE过程后可以快速分离目标分析物,提高了萃取效率。与已发表的测定三氯生的方法^[75]^相比,该方法有着方便快捷、耗时短等优点。

**图13:**
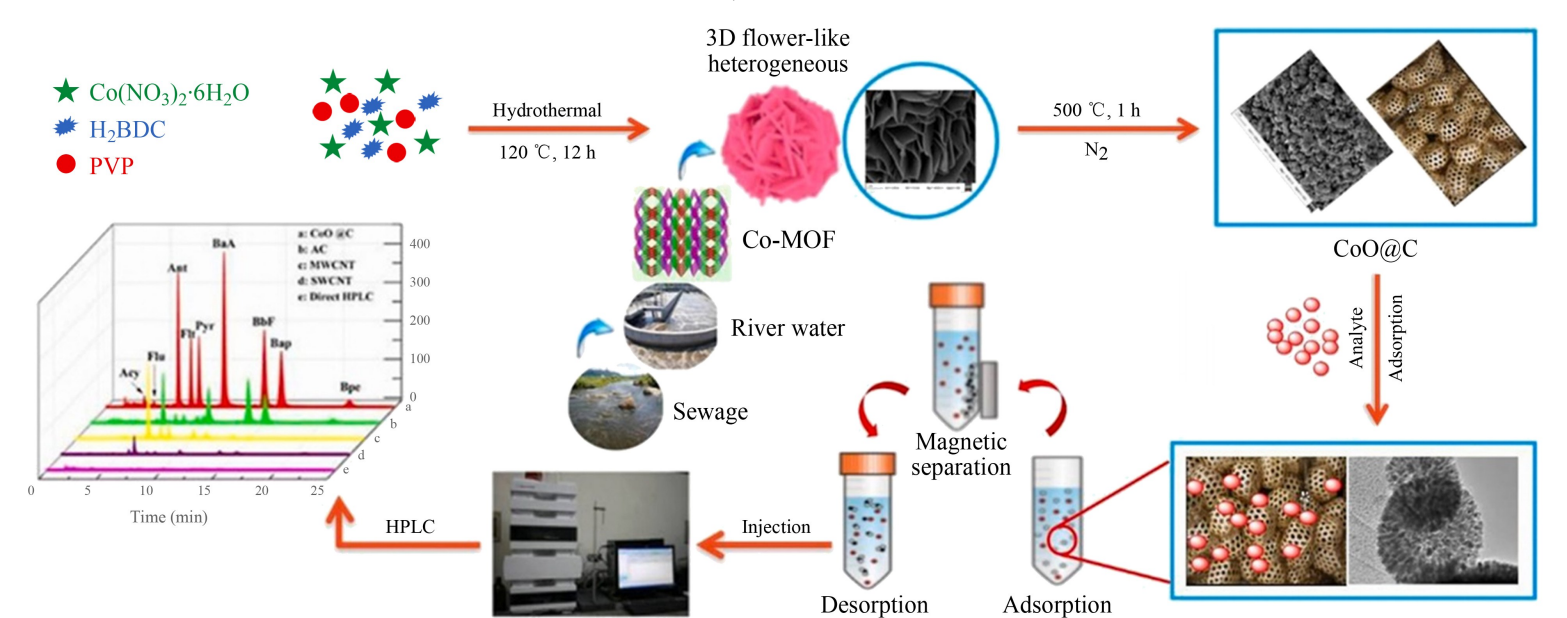
MSPE的示意图和CoO@C的制备过程^[70]^

### 3.4 分散固相萃取

DSPE是基于SPE发展起来的另一种新型样品前处理技术,该技术是由Anastassiades于2003年首次提出并成功应用在检测方向。与SPE技术不同的是,该种萃取技术在使用时所需要的有机溶剂量少,操作简便且由于目标分析物和吸附剂充分接触,萃取时间短,提高了萃取效率,因而广泛应用于食品、环境、农业、医药等领域^[76,77]^。DSPE的操作过程如下:将固体吸附剂材料加入到含有目标分析物的溶液中,固体吸附剂和目标分析物充分接触,目标分析物吸附到固体吸附剂上,然后通过离心或过滤等方法将目标分析物从样品溶液中分离,再用合适的有机溶剂对混合物进行洗脱,以此来富集目标分析物^[78,79]^。

DSPE技术常用的吸附剂主要有C18、氧化铝、活性炭、弗罗里硅土等,但是这些吸附剂的效果较差,对目标分析物不能做到特异性吸附,所以具有较好吸附性能的中空纳米材料近年来被广泛作为DSPE的吸附剂^[80,81]^。如Fu等^[82]^通过将硼氟化铵、叠氮化钠和硫混合在一起制备了具有超薄外壳的氮化硼空心球(BNHSs),将其用作DSPE的吸附剂,再结合GC-MS来测定环境水样中的痕量芳香污染物—多氯联苯(PCBs);该方法在0.15~250 ng/L范围内线性关系良好,检出限低(0.04~0.09 ng/L),重复性良好(RSD<12%, *n*=6),有较高的回收率(84.9%~101.0%)。用所建立的方法对河流、湖泊、雨水和泉水等真实环境样本进行分析,结果表明氮化硼空心纳米材料作为环境水样中有机污染物的吸附剂具有巨大的潜力。Jia等^[83]^通过溶剂热法成功制备了核壳纳米复合材料铟(Ⅲ)硫化物@金属-有机骨架(In_2_S_3_@MIL-125(Ti)),将其作为DSPE的吸附剂,并结合GC-MS来测定环境样品中的16种硝基多环芳烃(NPAHs)(图14);该方法在10 ~1000 ng/L范围内线性关系良好,检出限低(2.9~83.0 ng/L),重复性较好(RSD<10%, *n*=6),回收率为71.3%~112.2%,所建立的方法不仅具有较高的灵敏度,而且简单、快速、便捷,可用于测定食品和环境样品中的NPAHs。

**图14:**
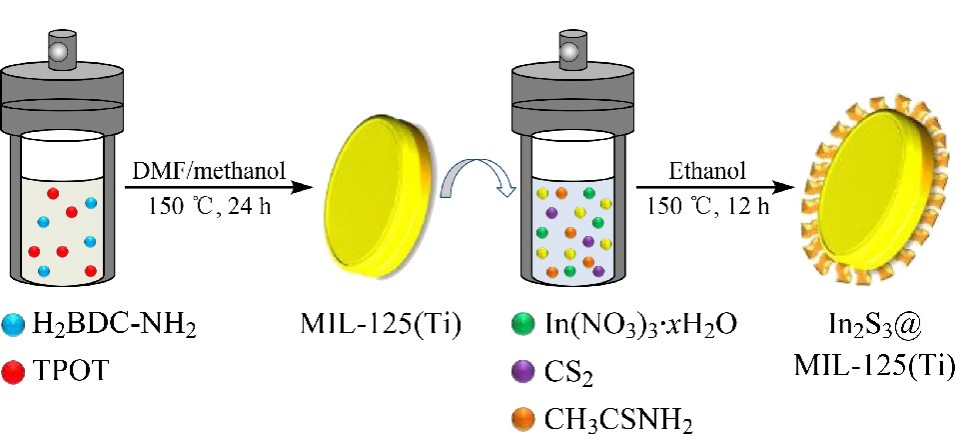
核壳纳米复合材料In_2_S_3_@MIL-125(Ti)的合成过程示意图^[83]^

综上,中空纳米材料在样品前处理中的应用见表1。

**表1:** 中空纳米材料在样品前处理中的应用

Method	Type of materials	Analytes	Combined method	Linear range	LODs	Ref.
SPE	CHPMs	V(Ⅴ), Cr(Ⅲ), Cu(Ⅱ), Cd(Ⅱ), Pb(Ⅱ)	ICP-MS	0.05-15 μg/L	0.8-3.2 ng/L	[47]
	NiCo_2_O_4_	Pb(Ⅱ)	FAAS	0.5-10 mg/L	5.9 μg/L	[48]
	h-PDA@L-lys	BR	HPLC	-	-	[49]
SPME	HCNCs	PAHs	GC-MS	5-2000 ng/L	0.03-0.70 ng/L	[57]
	Co_3_O_4_/C	PAHs	HPLC	0.005-1000 μg/L	0.002-2.680 μg/L	[59]
	Fe_2_O_3_/CeO_2_@MnO_2_	PAHs	GC-MS	0.04-100 μg/L	0.38-3.57 ng/L	[60]
MSPE	CoO@C	PAHs	HPLC	0.5-1000 μg/L	0.06-1.30 μg/L	[70]
	Fe_3_O_4_@C	PBDEs	GC-MS	1-1000 ng/L	0.07-0.17 ng/L	[72]
	Fe_3_O_4_@COFs	DPA	HPLC	0.1-100 mg/L	0.02-0.08 mg/L	[73]
	Fe_3_O_4_@MIL-100	triclosan	HPLC	0.1-50 mg/kg	0.03 mg/kg	[74]
DSPE	BNHSs	PCBs	GC-MS	0.15-250 ng/L	0.04-0.09 ng/L	[82]
	In_2_S_3_@MIL-125(Ti)	NPAHs	GC-MS	10-1000 ng/L	2.9-83.0 ng/L	[83]

DSPE: dispersive solid-phase extraction; CHPMs: hollow polymeric microsphere nanomaterials functionalized with carboxyl groups; h-PDA@L-lys: hollow structured nanomaterial L-lysine modified polydopamine coated shell; HCNCs: hollow carbon nanocubes; BNHSs: boron nitride hollow sphere; BR: bilirubin; PAHs: polycyclic aromatic hydrocarbons; PBDEs: polybrominated diphenylethers; DPA: diphenylamine; PCBs: polychlorinated biphenyls; NPAHs: nitro polycyclic aromatic hydrocarbons; ICP-MS: inductively coupled plasma mass spectrometry; FAAS: flame atomic absorption spectroscopy. - : Relevant data are not listed in the original literature.

## 4 结论与展望

随着纳米复合材料向功能化方向发展,设计满足不同要求的中空纳米材料具有重要意义。通过改变内核和外壳的结构或表面性质,可以获得许多具有特殊性质的中空纳米材料,并进一步拓宽其在样品前处理领域的应用价值。由于该种材料具有独特的中空结构、较大的比表面积、丰富的活性位点、制备方法多种多样以及结构功能多元化等优点,因而在样品前处理方面展现出了极大的应用潜力。然而,对于结构功能多元化的中空纳米材料而言,目前仅有少数的该材料应用于样品前处理领域,且它们对于目标分析物的吸附能力不是特别理想,因此提高中空纳米材料对目标分析物的吸附选择性是该材料在样品前处理领域发展的重中之重。为了实现对目标分析物的高效分离和富集,首先可以设计一些具有更大比表面积和优良孔径的中空纳米材料,以便对尺寸大小不一的目标分析物达到特定吸附;其次,将中空纳米材料与更多具有优异吸附性能的材料相结合,应用过程中发挥其各自的优势,实现材料之间的优势互补,使复合中空纳米材料具有更强的性能;此外还可以探索更多的绿色方法制备中空纳米材料,使其具有专一选择性,从而实现对目标分析物更好的特异性吸附。

现阶段,尽管中空纳米材料的合成已经取得了很大进展,但仍有一些问题需要解决。(1)对于软模板法,迄今为止所开发出来的方法在制备中空纳米材料时操作过程比较复杂、条件相对严格以及成本较高,不适合工业应用。因此我们有必要再探索一些相对比较简单且合成过程容易操作的、成本较低并适应于大批量工业生产的软模板法。(2)对于硬模板法,用该方法制备中空纳米材料时需要额外的模板添加剂或者高温煅烧步骤来去除模板,但在该过程中使用到的部分化学物质有毒且对环境有害;在高温煅烧时,如果温度、升温速率等因素控制不当,可能会对中空结构造成不同程度的影响。因此,这种方法制备中空纳米材料时有着成本高、复杂,并且涉及剧毒物质等缺点,目前最重要的是找到合适的解决方案来克服这些缺点。(3)对于自模板法,合成中空纳米材料时也有一些难题急需改进。例如,用蚀刻法来制备中空纳米材料时,虽然表面保护在某些情况下可能起作用,但它在很大程度上取决于外部环境,因此如何确保蚀刻是优先发生在模板的内表面,是目前面临的一个巨大挑战。

总之,随着研究人员对纳米材料不断探索和研究,必将会有更多性能优异的中空纳米材料应用于样品前处理领域,样品种类和分析对象将会更加广泛,应用前景也会更加广阔。同时,科学技术也在不断地发展进步,中空纳米材料在前处理领域的应用还有更多可探索的空间。
